# Electrochemical Performance of Engineered NiCo_2_O_4_ in AEM Water Electrolyzers: Direct-Growth vs Spray-Coated Anode

**DOI:** 10.1021/acsaem.5c01487

**Published:** 2025-08-05

**Authors:** Ataollah Niyati, Arianna Moranda, Sebastiano Bellani, Thi Hong Hanh Le, Michele Ferri, Ombretta Paladino

**Affiliations:** † Department of Civil, Chemical and Environmental Engineering (DICCA), Università di Genova, Via Opera Pia 15, 16145 Genoa, Italy; ‡ Antares Electrolysis S.r.l., Piazza della Vittoria, 14/19, 16121 Genova, Italy; § Nanochemistry Department, Istituto Italiano di Tecnologia (IIT), Via Morego 30, 16163 Genova, Italy

**Keywords:** anion exchange membrane water electrolysis, alkaline water electrolysis, green hydrogen production, oxygen evolution reaction, NiCo_2_O_4_, sono-hydrothermal method, binder-free

## Abstract

This study investigates the synthesis and electrochemical performance of NiCo_2_O_4_ anodes for water electrolysis in both alkaline and anion exchange membrane (AEM) configurations. An engineered direct growth method using a urea-mediated sono-hydrothermal approach was used to synthesize NiCo_2_O_4_ on Ni felt, creating a binder-free electrode optimized for an alkaline environment. We evaluated the electrode’s performance in AEM water electrolysis, comparing it with a spray-coated electrode incorporating ionomers and the same electrocatalyst. Our findings highlight that direct-grown binder-free electrodes, produced through varied synthesis routes, exhibit remarkable activity and stability in AEM cells operated in dry cathode mode (1.90 V @ 1 A cm^–2^), with seamless interaction between the catalyst layer and the membrane. Moreover, this binder-free NiCo_2_O_4_ on Ni felt is also an efficient anode under alkaline electrolysis configuration, exhibiting high stability and remarkable performance (1.78 V @ 1 A cm^–2^, 1.92 V @ 2 A cm^–2^), ascribable to the increased conductivity and improved charge transfer resistance of the catalyst layer.

## Graphical abstract

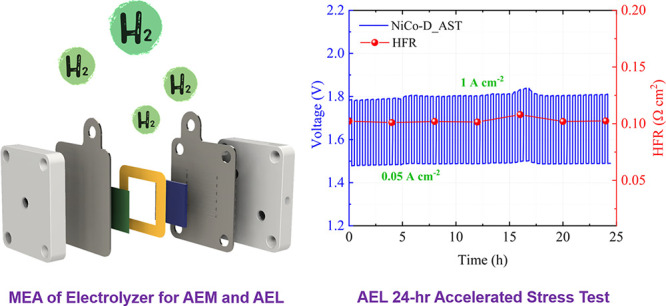

## 1 Introduction

In today’s world, sustainable energy solutions have brought attention due to the importance of not only hydrogen production technologies but also alternative fuel synthesis.^1^ To this end, water electrolysis stands out as a reliable approach for producing green hydrogen for achieving zero emissions by serving as a clean fuel or, most likely, as a chemical feedstock.^2,3^ Among several electrolysis setups, alkaline water electrolysis seems to be an efficient and well-established technology due to its unique characteristics such as simplicity, low cost, and high stability under operation.^4,5^ However, alkaline water electrolyzers (AELs) face several drawbacks when paired with intermittent renewable energy sources, such as wind and solar power. These include: (i) slow dynamic response, especially under atmospheric pressure, which can limit the efficiency and result in inconsistent hydrogen production^6^; (ii) need for implementing strategies to maintain a continuous operation or partial load, operation through complementary storage units, as frequent start–stop cycles caused by the variable output of renewables and associated with temporary reverse polarization can lead to mechanical stress and degradation of AEL components, particularly electrodes^7,8^ ; (iii) limited part-load efficiency since at lower loads, excessive gas crossover can occur, compromising safety and potentially leading to shutdowns, which further limits flexibility in fluctuating power conditions.^9–11^

On the one hand, anion exchange membrane water electrolyzers (AEM-WEs) and proton exchange membrane electrolyzers (PEM-WEs) are generally considered to be more resilient when operated with intermittent energy sources due to their quicker dynamic response, higher efficiency at part-load, and better adaptability to fluctuating power inputs, making them more compatible with renewable energy sources than conventional systems such as alkaline electrolyzer.^12–15^ They have a higher power density and can also operate under differential pressure, making them ideal for reducing postproduction costs associated with hydrogen gas compression. In this context, AEM-WEs combine several advantages of both AELs and PEM-WEs, while offering additional benefits, such as compatibility with a broader range of catalysts and higher operating flexibility. In particular, AEM-WEs do not necessarily require the presence of precious metal catalysts such as Pd and Pt, or allow the reduction of the load of these metals, significantly decreasing overall system costs while (ideally) eliminating the need for several critical raw materials.^16–18^ Despite these benefits, the success of AEM-WE technology is still linked to the understanding of the electrode–membrane interface and the role of ionomers in facilitating efficient ion transfer.^19–21^ The presence of ionomers in AEM-WE fed with pure water seems to be essential, as they are the only ionic conductors.^22^ Conversely, when operating AEM-WEs with alkaline electrolytes (e.g., 1 M KOH), binder-free electrodes could show better performance, as the absence of ionomers avoids blockage of the catalyst active sites and the decrease of electronic conductivity of the catalyst layer. However, the overall impact of ionomers on cell stability and efficiency needs further investigation.^22^

In this study, we focus on the performance assessment of morphology-engineered NiCo_2_O_4_-based anodes produced as both freestanding, binder-free electrodes, obtained by direct growth onto the substrate, and their ionomer-containing counterparts, obtained by spray-coating. Both electrodes have been tested in zero-gap AEL and AEM-WE configurations. NiCo_2_O_4_ is recognized for its excellent electrochemical activity in oxygen evolution reactions (OER), due to its chemical stability and relatively low cost, making it a strong candidate for integration into commercial electrolysis systems.^23–26^ To synthesize NiCo_2_O_4_, we use the sono-hydrothermal approach, a versatile technique that enables the production of highly crystalline nanostructures with controlled morphology and dispersion to achieve nanorods with particle size between 20 to 45 nm.^27,28^ In particular, the sono-hydrothermal process allows for the direct growth of NiCo_2_O_4_ on Ni felt, creating a binder-free, high-surface-area electrode specifically designed for enhanced performance in AELs.^29^

However, given the promising attributes of AEM-WEs, we sought to extend the application of our engineered NiCo_2_O_4_-based anodes to AEM-WEs, where the presence of an ionomer in the electrodes and the engineering of catalyst-membrane interfaces typically play a central role in determining the overall cell performance. To this end, we prepared NiCo_2_O_4_-based electrodes through both direct growth and spray-coating techniques, with the latter involving the use of ionomers that may enhance the electrode/AEM interactions by establishing continuous anion-conducting pathways. Spray-coating is a widely adopted technique for the fabrication of AEM-WE electrodes, allowing for the precise control of catalyst layer thickness and distribution over the substrate surface.^30–33^ In this work, NiCo_2_O_4_ powders synthesized through the sono-hydrothermal method were formulated into inks containing an ionomer and subsequently spray-coated onto the Ni felt substrates. Through comparative analysis of the two fabrication techniques, we aim to elucidate the influence of ionomer on the electrode performance and stability in both AEL and AEM-WE cells.

## 2 Experimental Section

### 2.1 Materials

NiCl_2_·6H_2_O (99% purity, Carlo Erba Reagents, Italy) and CoCl_2_·6H_2_O (98% purity, Carlo Erba Reagents, Italy) served as Ni and Co precursors. KOH (99% purity, Carlo Erba Reagents, Italy) and urea (99% purity, Carlo Erba Reagents, Italy) were used for the electrocatalyst synthesis as well as the electrolyte source. Additionally, 10 wt % Nafion dispersion (D1021 Nafion, Fuel Cell Store, USA) and Zirfon Perl UTP 220 diaphragm (Agfa) were adopted for AEL; while Aemion+ AF3-HWK9-75-X AEM and related ionomer (Ionomr Innovations, Canada) and Pt/C (30 wt %, from Cabro S.p.A., Italy) were used in AEM-WE cells. Ethanol (EtOH), 2-propanol (IPA) were purchased from Sigma-Aldrich (Germany). Ni felts (99% pure nickel, 1 mm thickness, porosity 0.85) gas diffusion layers supplied from QL Metal Fiber Co (China) were used to fabricate the electrodes.

### 2.2 Direct-Growth Preparation of Electrodes for the OER

The engineered catalyst and electrodes are produced by following the methodology described in Niyati et al.^34^ The direct growth method is a binder-free method to coat directly the electrocatalyst (NiCo_2_O_4_) onto the surface of the Ni felt, as illustrated in Figure 1. Experimentally, Ni felt was first immersed in a solution of 3 M HCl for 5 min, followed by rinsing in a mixture of Milli-Q water and acetone for 15 min with sonication. After cleaning, it was dried overnight in a vacuum oven at 60 °C. The dried Ni felt was weighed before and after the sonothermal process so as to accurately determine the amount of catalyst loaded on the electrode through the direct growth method.

**Figure 1:**
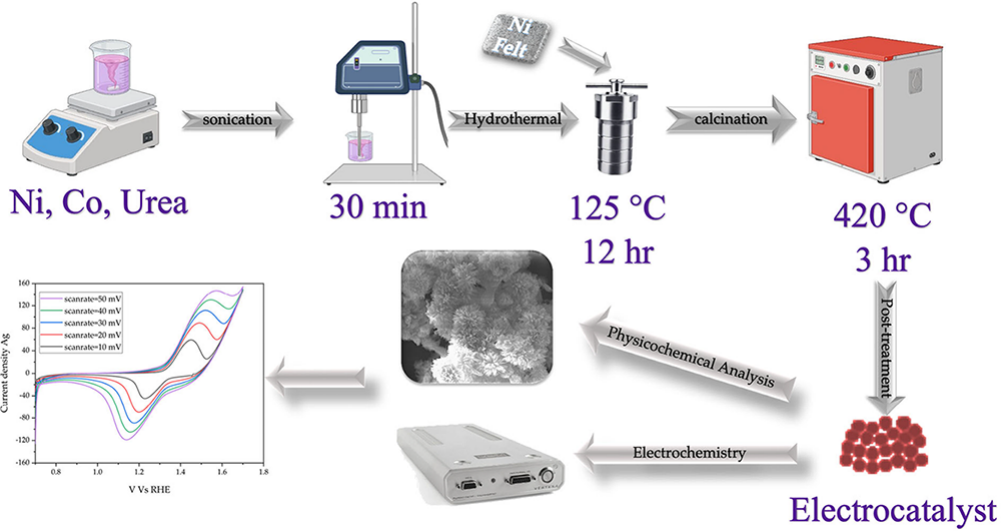
Synthesis of NiCo_2_O_4_ powder and fabrication of the NiCo-D electrode via a urea-mediated sono-hydrothermal process.

The NiCo_2_O_4_ electrocatalyst was synthesized using a design of experiments (DOE) approach to optimize the operating conditions of the sono-hydrothermal process and using urea as a hydrolysis agent, as described in Paladino et al.^27^ To synthesize the NiCo_2_O_4_ electrocatalyst, 2.015 g of NiCl_2_·6H_2_O was dissolved in 25 mL of Milli-Q water and slowly added dropwise to a CoCl_2_·6H_2_O solution containing 4.0354 g of Co precursor in 40 mL of Milli-Q water. In the second step, urea was added in a 1:10 molar ratio, and the solution was vigorously stirred for 30 min. To enhance nucleation and nanoparticle formation, the solution was subjected to sonication at 100 W for an additional 30 min. The resulting solution was then transferred to a 100 mL Teflon-lined stainless-steel autoclave for the hydrothermal reaction. Clean Ni felt was placed inside the autoclave, and the reaction was carried out at 125 °C for 12 h. After the completion of the reaction, the electrode was thoroughly washed with Milli-Q water and ethanol to remove any residual impurities or excess NiCo_2_O_4_. The powder of NiCo_2_O_4_ was collected by filtration and washed with ethanol and Milli-Q water to be used for spray coating. Both the collected powder and electrode were then dried overnight in a vacuum oven at 60 °C. In the final step, the dried powder and Ni felt underwent thermal treatment in air at 420 °C for 3 h with a heating ramp of 10 °C/min. This process ensured the successful synthesis of NiCo_2_O_4_ with the desired characteristics for subsequent analysis and application. Through this process, nucleation of NiCo_2_O_4_ particles, which lead to nanorod structures, occurred directly on the active surface of the Ni felt, achieving a catalyst loading of approximately 5 mg/cm^2^, which is confirmed by weighing the bare Ni Felt before sono-hydrothermal reaction and after calcination. This so-produced electrode is hereafter named NiCo-D.

### 2.3 Spray-Coating Preparation of the Electrode

The spray coating method was selected as an established and widely known technique for the fabrication of electrodes for AEM-WE.^4,33,35–37^ The NiCo_2_O_4_ ink was prepared by mixing the catalyst powders with Aemion+ AF3-HWK9-75-X ionomer (for AEM-WE) or Nafion binder (for AEL, to withstand strongly alkaline media) in an EtOH:water mixture 1:1,^38^ using a catalyst concentration from 2 to 5 g/L and ionomer concentration from 0.2 to 1 g/L,^31^ i.e., in the optimal ranges suggested by Faid et al.^39^ The dispersion was then homogenized by sonication for 1 h before use. The catalyst ink was sprayed using a spray gun with a 0.5 mm nozzle (FE-134K aerograph plus FD-186 compressor, Fengda, operated at 1.5 bar with compressed air) onto the Ni felt, with the substrates placed on a hot plate at 80 °C.^39,40^ The amount of deposited catalyst was controlled by weighing the electrodes before and after catalyst deposition until reaching a catalyst loading of 5 mg/cm^2^ to have the same loading as that of the direct-grown electrode NiCo-D, and the electrode is hereafter named NiCo-S. The catalyst load was the same for both directly grown and sprayed ones.

The Pt/C inks, used for the preparation of cathodes, were prepared similarly to the NiCo_2_O_4_ ones and adjusting the ionomer concentration as suggested in the work of Koch et al.,^31^ ranging from 0.2 to 0.3 g/L. The Pt mass loading was 0.5 mgPt/cm^2^, compliant with standard low PGM cathodes.^41,42^

### 2.4 Physicochemical Characterization of Materials

Various analytical techniques were used to thoroughly investigate the physical and chemical properties of the materials. X-ray diffraction (XRD) measurements were conducted using a PANalytical AERIS diffractometer, allowing analyzing the crystal structure and phase purity of the samples. The morphology of the samples was investigated with a TESCAN scanning electron microscope (SEM), which is coupled with energy-dispersive spectroscopy (EDS) was utilized to determine both the distribution and elemental composition of the materials and electrodes used in this study. Additionally, transmission electron microscopy (TEM) was performed with a JEM 2100 Plus instrument from JEOL Ltd. (Japan) to reveal the morphology and structure of the NiCo2O4 powder.

### 2.5 Evaluation of Electrochemical Performances

Three-electrode cell measurements were performed using an IVIUM Vertex 10A potentiostat workstation (Ivium Technologies B.V., Netherlands), and a 1 M aqueous KOH was used as the electrolyte. The working electrodes (1 cm × 1 cm) included NiCo-S, NiCo-D, and Ni felt (the latter representing the operative blank). A Hg/HgO, filled with a 1 M KOH solution, was used as the reference electrode, while the counter electrode was a Pt foil (0.5 mm ID, BAS, Japan). All the potentials reported in the paper are converted to the RHE scale and corrected by the ohmic drop of the system by usingeq [Disp-formula eq1]:

$$E(\mathrm{RHE})=E_{\mathrm{Hg}/\mathrm{HgO}}+0.927\;V-iR_{\mathrm{uc}}$$ 1

where *R*_uc_ is the series resistance calculated from EIS.^43,44^ Polarization curves were recorded through backward linear sweep voltammetry (LSV) in the range of 1.0 to 1.7 V vs RHE at a potential scan rate of 5 mV/s for better estimation of the OER overpotentials and activity. Backward LSV allows avoiding the influence of oxidation state changes that typically occur during the forward scan, which can affect the accurate determination of overpotentials at lower current densities (e.g., 10 mA cm^–2^). Electrochemical impedance spectroscopy (EIS) was performed across a frequency range of 0.01 to 100,000 Hz at a potential of 0.55 V vs Hg/HgO. The obtained spectra were matched and fitted with the proper equivalent circuit (represented mostly by *R*(*RQ*)) to regress *R*_uc_.

A VMP3 potentiostat (Biologic, France) equipped with an external 20 A booster channel and an external EIS channel was used to power water electrolyzer single cells, performing galvanostatic polarization curves and EIS measurements. Polarization curves were collected galvanostatically according to a stepwise chronopotentiometric approach. The current density window investigated ranged from 50 mA cm^–2^ to 2 A cm^–2^, with each step lasting 3 min. *I*–*V* data were obtained by averaging the cell voltage obtained from the last minute of each step. High-frequency resistance (HFR), associated with the separator resistance from the intercept of the Nyquist plot with the real axis at 100,000 Hz, was recorded to evaluate the change in MEA. It is important to note that all the data for the cells (AEL and AEM-WE) are presented without i × HFR correction, reflecting the practical operating conditions typically encountered in industrial applications. No HFR correction was applied to the zero-gap cell, as these tests were designed to reflect industrially relevant conditions where ohmic losses, mainly from MEA, are intrinsic to the system. In contrast, *iR* correction was applied to the three-electrode measurements, following standard practice in electrocatalysis, to more accurately assess the intrinsic activity and stability of the catalysts.

### 2.6 AEL and AEM-WE Single Cell Assembly

The AEL and AEM-WE (with an active area of 5 cm^2^) single cells were assembled using the zero-gap cell fixture produced by Antares Electrolysis (Italy), which includes corrosion-resistant Ni anodic plates equipped with flow fields and Ethylene-Propylene Diene Monomer O-ring seals. The cell components, including the separator (Zirfon Perl UTP 220 diaphragm for AEL and Aemion+ AF3-HWK9-75-X for AEM-WE) and electrodes, were compressed to achieve zero-gap cell configurations. In the case of AEM-WE cells, the assembly was performed using activated and hydrated AEMs to prevent cracking and damage to the membranes.^45^ Moreover, the membrane is activated by placing it inside the 1 M KOH for at least 24 h. For each electrode compartment, polytetrafluoroethylene spacers were used to ensure proper compression of the cathode/AEM/anode stack (i.e., the MEA). The spacer thickness was optimized to ensure a GDL compression of about 15% ± 5%. Sprayed Pt/C electrodes were used as cathodes, while NiCo-D or NiCo-S were used as anodes, and all electrodes, before mounting inside the cell, were kept in 1 M KOH for at least 30 min. The cells were operated differently depending on the separator used. AEL cells were operated at 80 °C using 30 wt % aqueous KOH as electrolyte, flowing at both anode and cathode. Two different reservoirs were used for the anolyte and catholyte, both flowed at 5 mL min^–1^. AEM-WE cells were operated at 60 °C using 1 M aqueous KOH as electrolyte in dry cathode mode. Therefore, the electrolyte was fed only to the anode with a flow rate of 5 mL min^–1^. Both types of cells were operated in a two-electrode configuration. For AEM-WE, Aemion+ AF3-HWK9-75-X ionomer is used as the binder for spraying Pt/C on the cathode as well as NiCo-S on the anode. For AEL, the Pt/C is sprayed using Nafion binder (with a binder to catalyst weight of 2:10), corresponding to a weight ratio. Nafion is used for spraying AEL's cathode since it is adopted for AEL at an industrial scale, due to its good stability and lower cost than that of the anionic binders.

## 3 Results and Discussion

### 3.1 Physicochemical Characterization of Materials

The XRD, SEM, EDS, and TEM characterizations were performed to evaluate the physicochemical properties of the synthesized materials and electrodes, as shown in Figure 2.

**Figure 2:**
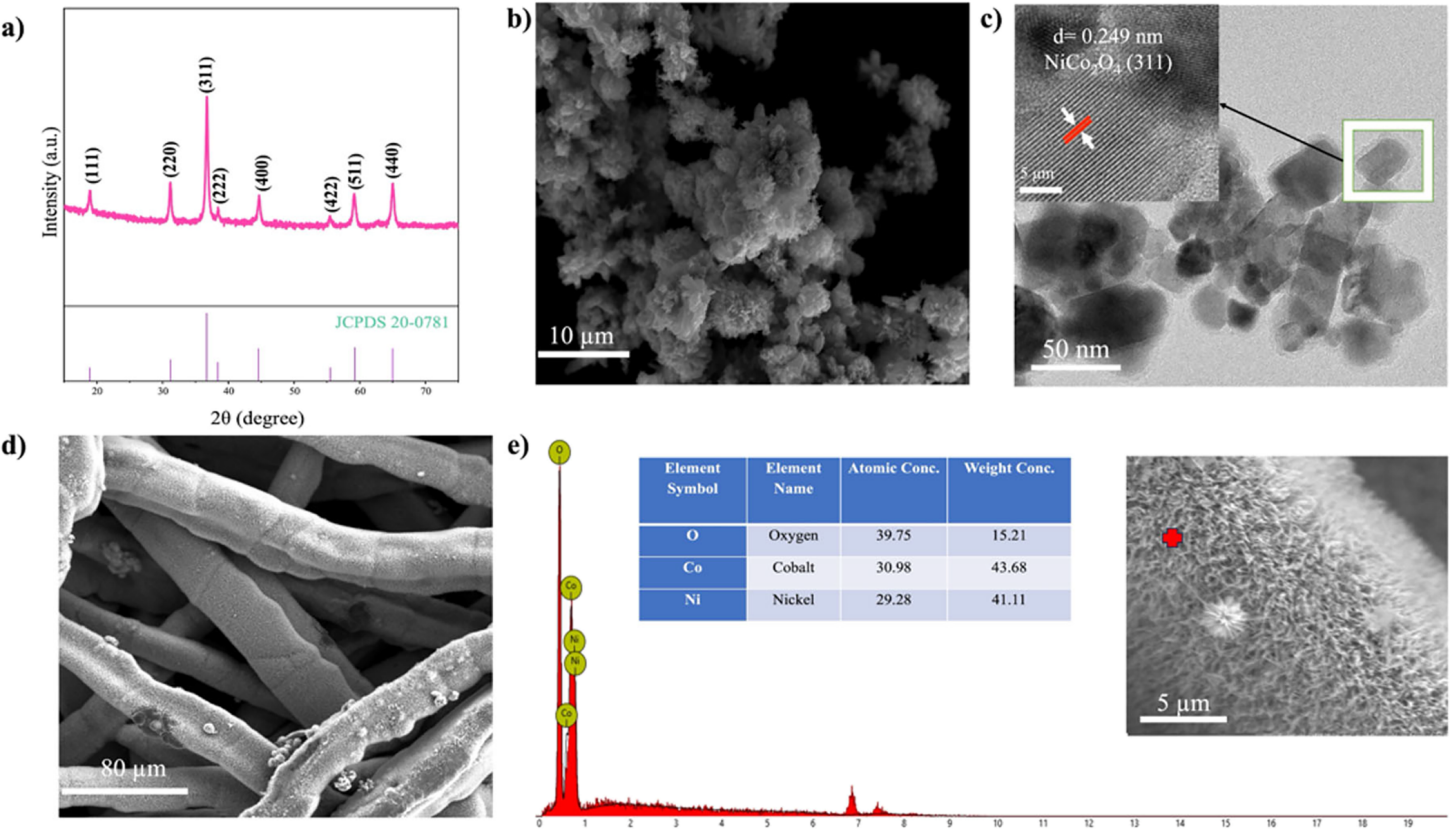
Physicochemical characterization of materials: (a) XRD of NiCo_2_O_4_. (b) SEM of NiCo_2_O_4_ collected on the powder sample obtained by the sono-hydrothermal method. (c) TEM image collected on the powder NiCo_2_O_4_ sample obtained by the sono-hydrothermal method. (d) SEM image and (e) EDS spectrum and related image (spectrum collected in the red spot), on the pristine NiCo-D electrode.

Figure 2a shows the XRD pattern of the synthesized NiCo_2_O_4_ powder within the 2θ range of 20° to 80°. According to JCPDS No-20-0781, the peaks observed at 18.90°, 31.15°, 36.70°, 38.40°, 44.62°, 55.43°, 59.09°, 64.98°, and 77.54° correspond to the (111), (220), (311), (222), (400), (422), (511), (440), and (533) crystallographic planes, respectively, confirming the cubic structure of NiCo_2_O_4_.^46^ The sharp, well-defined peaks indicate high crystallinity, meaning that the sono-hydrothermal method combined with calcination fosters optimal nucleation of the electrocatalyst. This is further corroborated by SEM and TEM analyses of the NiCo_2_O_4_ powder, as shown in Figure 2b,c, respectively. Figure 2b displays an SEM image revealing a mum-flower-like structure, where the NiCo_2_O_4_ rods cluster together. The TEM analysis in Figure 2c reveals particle sizes in the range from 20 to 45 nm. Additionally, the lattice spacing (*d*) of 0.249 nm, associated with the (311) plane of NiCo_2_O_4_ spinel, validates the successful synthesis via the sono-hydrothermal method, with urea serving as the hydrolysis agent.^47^

The electrode fabricated using the direct growth method (NiCo-D) was investigated through SEM and EDS measurements, as reported in Figure 2d,e, respectively. Figure 2d reveals a homogeneous growth of NiCo_2_O_4_ electrocatalyst across the Ni felt fibers while maintaining the porosity of the electrode and preventing blockage of the pore sites. The rod-like structure of the electrocatalyst, together with its nanostructuring, provides a large surface area, aiming at enhancing electrochemical reaction kinetics.^48,49^ The EDS analysis in Figure 2e further confirms the purity of NiCo_2_O_4_ and aligns with the expected stoichiometry of the spinel structure. The use of urea as a hydrolysis agent, combined with the sonothermal synthesis method, enables the formation of small NiCo_2_O_4_ nuclei. These nuclei grow homogeneously not only on the surface of the activated Ni felt but also inside the pores (the detail in Figure 2e shows the single fiber of NiFelt with NiCo-D grown on it), ensuring an even distribution of Ni^2+^ and Co^2+^ ions and contributing to the electrochemical activity of the electrocatalyst; however the presence of electrocatalyst is limited at the bottom of the GDL (Figure S7 in SI).^50,51^

Overall, XRD, SEM, and TEM analyses confirm the successful synthesis of the NiCo_2_O_4_ spinel electrocatalyst via the sonothermal method using urea as a hydrolysis agent. XRD demonstrates high crystallinity, while SEM and TEM analysis reveal mum-flower-like rod structures with high purity.

### 3.2 Assessment of Electrodes OER Performance in a Standard Three-Electrode Setup

Three-electrode cell configuration experiments were carried out on NiCo-D, NiCo-S, and bare Ni felt to preliminarily assess their catalytic activity for the OER before being moved to industrially relevant AEL or AEM-WE testing in a two-electrode flow cell configuration.

Figure 3a reports the 100% *iR*_uc_-corrected LSV curve, acquired using the backward potential scan mode. NiCo-S, NiCo-D, and Ni felt achieved a current density of 10 mA cm^–2^ at 1.544, 1.531, and 1.627 V vs RHE, which corresponds to 314, 301, and 397 mV of overpotentials (Figure 3b), respectively. Clearly, the presence of NiCo_2_O_4_ on the surface of Ni felt significantly improves the electrochemical activity of the bare support, regardless of the electrode fabrication method (i.e., whether a binder is used or not). Tentatively, this higher activity of the freestanding NiCo-D electrode is ascribed to the absence of the binder in the catalyst layer, resulting in a higher number of OER active sites exposed to the electrolyte.

**Figure 3:**
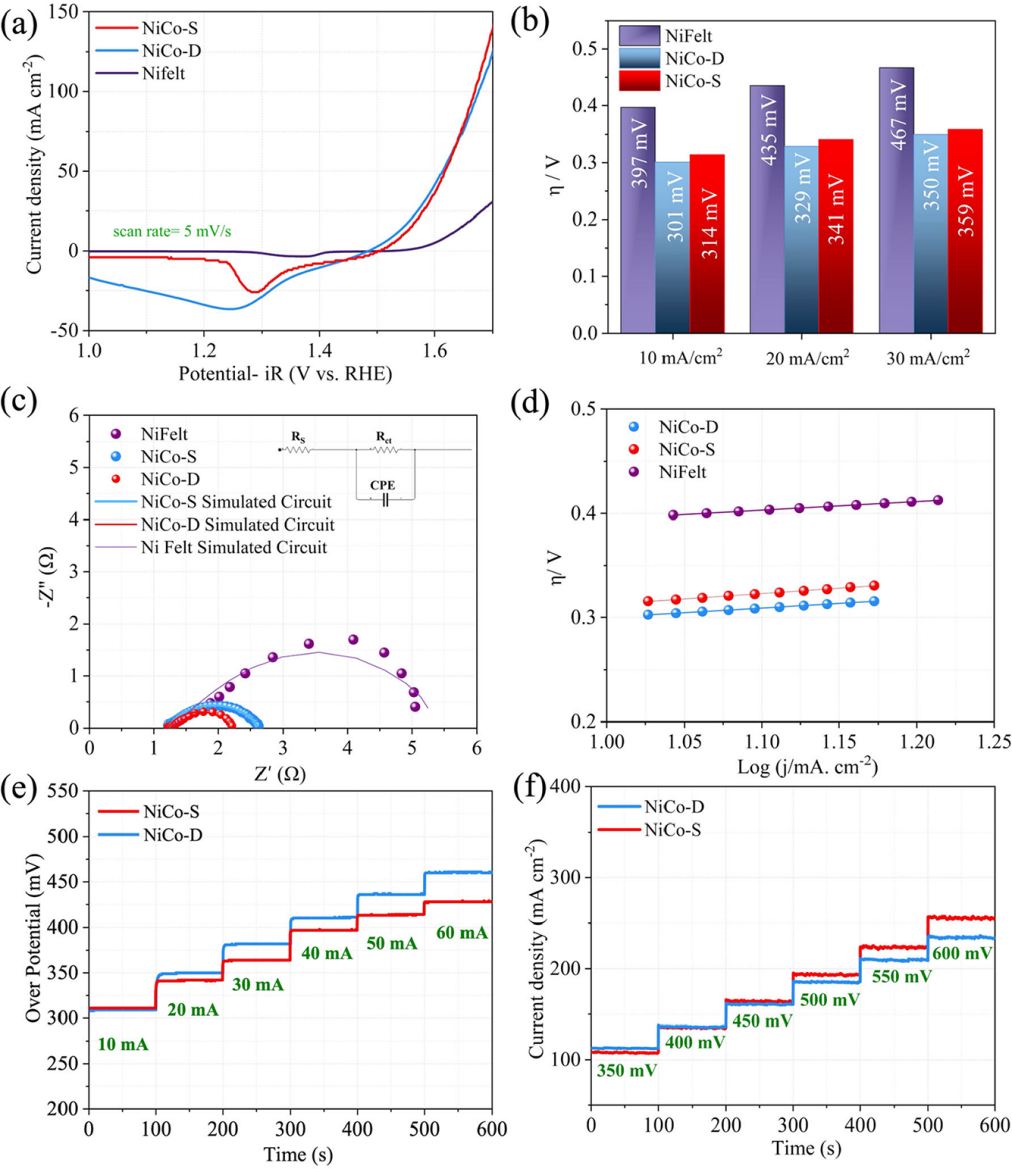
Three-electrode cell configuration measurements for NiCo-D, NiCo-S, and Ni felt: (a) backward LSV scans, with 5 mV/s scan rate (b) comparison of the OER overpotentials measured for the electrodes at different current densities. (c) Electrochemical impedance spectroscopy (EIS) analysis and (d) pseudo-Tafel plots for the investigated electrodes. (e) Chronopotentiometry (CP) and (f) chronoamperometry (CA) tests were collected at different current and potentials (CP and CA reported without *iR*_uc_ correction).

As shown in Figure 3c, Nyquist plots were fit using an electrical equivalent circuit, shown in the inset panel. Here, *R*_uc_ is the uncompensated resistance of the system, at high frequencies (∼1.23 Ω at 100 kHz for all of the investigated electrodes extracted from the equivalent fitted circuit), and *R*_ct_ is the charge transfer resistance, corresponding to the diameter of the semicircles. Estimated *R*_ct_ are 1.38, 0.996, and 3.53 Ω for NiCo-S, NiCo-D, and Ni felt, respectively. Consistent with previous results and considerations, the lower *R*_ct_ is measured for the freestanding NiCo-D electrode, for which the electrocatalyst is directly grown on the Ni felt in the absence of any ionomer/binder, thus facilitating the transfer of electrons. On the other hand, the NiCo-S, which is fabricated by spraying an electrocatalyst ink (containing a 1:10 solid weight ratio of binder to catalyst inside the ink) on the surface of the Ni felt, displays a higher *R*_ct_.

Figure 3d shows the pseudo-Tafel slopes for NiCo-S, NiCo-D, and Ni felt extracted from LSV with a 5 mV/s scan rate and a 2 mV step, providing key information about the OER kinetics of the electrodes (the description of pseudo-Tafel slopes is provided in the SI). The slopes measured for NiCo-S, NiCo-D, and Ni felt are 84.1, 79.7, and 130.4 mV/dec, respectively, consistently demonstrating that NiCo-D exhibits enhanced OER kinetics with respect to the other electrodes. It is also noteworthy that the presence of NiCo_2_O_4_ on the support results in a significant enhancement of its electrochemical activity compared with bare Ni felt.

The OER activity of both NiCo-S and NiCo-D was further evaluated by means of either chronopotentiometry (CP) or chronoamperometry (CA) protocols, also aimed at a preliminary assessment of their stability. In the CP and CA test (Figure 3e,f), both protocols consistently indicate that NiCo-D performs better at low current densities while NiCo-S works better at higher current densities, consistent with the LSV. This behavior could be attributed to a slightly different gas bubble removal capability of the two electrodes, more noticeable at higher current densities for gas bubble detachment (the catalyst layer on NiCo-D is in a 3D structure, while in NiCo-S the catalyst is present only on the surface, due to spraying on GDL). These data highlight once more how an electrocatalyst, deemed the most promising from most three-electrode testing at lab-scale, might turn out to be less performant under industrially relevant conditions, for example, when required to operate at high current densities. With the aim of validating our electrodes in a real industrial scenario, we then proceeded to test them in flow cells under both AEL and AEM configurations.

#### 3.2.1 AEL Test of the Direct-Growth Anode

Binder-free electrodes offer significant advantages for AELs, particularly in terms of durability and efficiency.^52^ By eliminating binders, which can degrade in harsh alkaline environments, these electrodes reduce the risk of catalyst detachment and ensure long-term stability. Moreover, the direct integration of the catalyst into the substrate enhances electrical conductivity by providing uninterrupted pathways for electron transfer, optimizing the performance. Additionally, binder-free designs simplify manufacturing processes, lowering costs and making them more suitable for large-scale applications. Based on these considerations, NiCo-D was first tested as an anode in a 5 cm^2^ zero-gap AEL single cell fed with 30 wt % KOH aqueous electrolyte and operating at 80 °C. A Pt/C electrode (see the Methods section for details) was used as a benchmark cathode. At first, the full cell underwent an activation procedure (mainly needed to condition the diaphragm and the electrodes but also to allow for the thermalization of the overall system), which is reported and commented in the Supporting Information, Figure S1. Briefly, the *I*–*V* curve of the cell stabilizes in the last 30–40 min of the activation process (ca. 3 h long), with the cell voltage plateauing at ca. 1.78 V (@ 1 A cm^–2^) and the HFR of the system reaching a stable minimum value at ca. 0.1 Ω cm^2^. The activation was followed by the collection of a galvanostatic polarization curve (blue stars in Figure 4a), a 24 h long CP step at 1 A cm^–2^ (Figure S2), a 24 h accelerated stress test (AST, Figure 4b), and a final galvanostatic polarization curve (blue circles in Figure 4a). The initial polarization curve demonstrates that the AEL implementing NiCo-D achieves state-of-the-art performance, delivering 1 and 2 A cm^–2^ at 1.78 and 1.96 V, respectively. The stability of the performance at 1 A cm^–2^ is confirmed by the day-long CP registered afterward, with minimal potential oscillation, mainly imputable to electrolyte refilling operations and temperature variations in the lab (Figure S2, blue curve). Consistently, the HFR (red circles in Figure S2) is stable and low throughout the whole experiment. The AST corroborates the stability data, demonstrating that the cell can withstand harsh load variations (cycling from 1 A cm^–2^ to 50 mA cm^–2^ every 15 min for 24 h) and thus is, in principle, compatible with renewable energy sources. It is noteworthy that the AEL cell not only maintained this performance after the AST (post-AST polarization curve, Figure 4a, blue circles) but also achieved better performances, showing 1.73 V at 1 A cm^–2^ and 1.92 V at 2 A cm^–2^. Such an increase is tentatively ascribed to the roughening of the electrode under harsh alkaline and anodic conditions, leading to an increase in the physical and electrochemically active surface area. Overall, these results highlight the remarkable OER activity of the NiCo-D electrodes and the stability of the present AEL cell configuration and the embedded catalysts.

**Figure 4:**
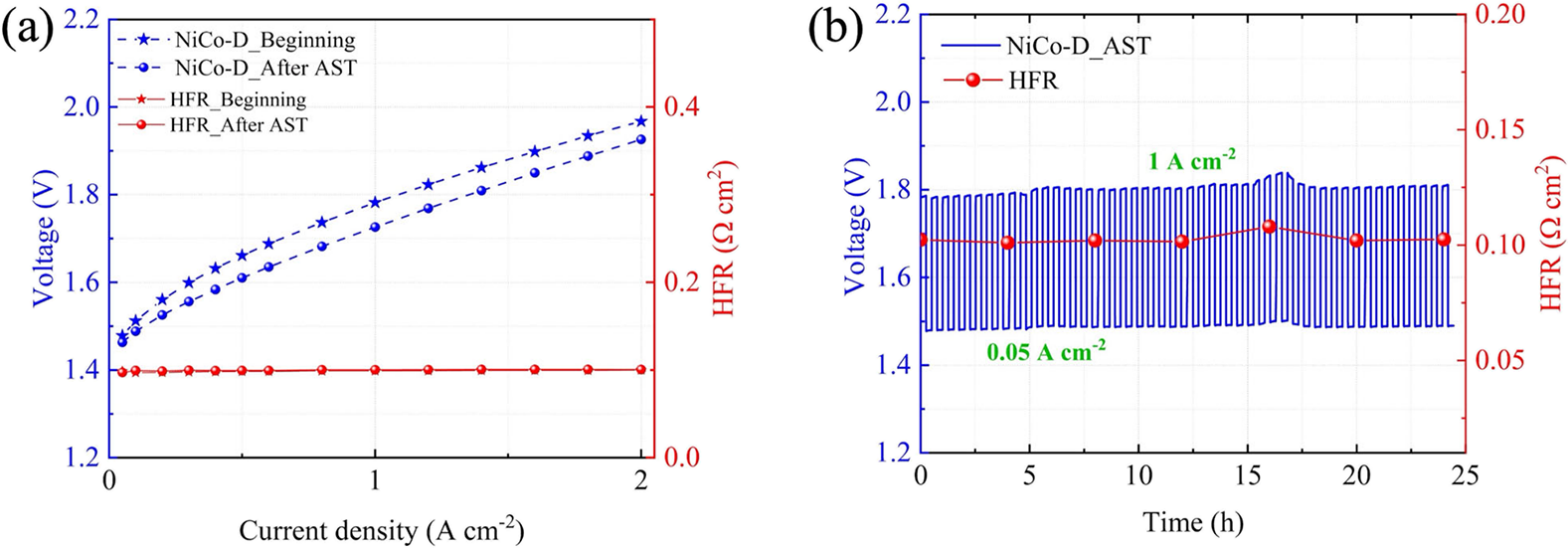
Characterization of a single cell AEL implementing NiCo-D as the anode: (a) Galvanostatic polarization curves (and HFR from EIS at each galvanostatic step) collected before (stars) and after (circles) the AST. (b) 24-h-long AST.

#### 3.2.2 AEM-WE Test of Direct-Growth and Spray-Coated Anodes

In AELs, binder-free electrodes are highly valued for their simplicity and stability. However, under the AEM-WE configuration, electrodes require the presence of ionomers in the catalyst layer to ensure ionic continuity and thus achieve optimal performance. Nonetheless, ionomers can reduce the electrode activity by blocking access to active sites. Currently, the role of ionomers in AEM-WEs is still under study.^20,53^ Although binder incorporation in the cathode layer is paramount in AEM-WE operated in dry cathode mode, there are markedly fewer investigations dedicated to shedding light on the importance of ionomers in the anodic catalyst layer.

In order to investigate this matter, NiCo-D and NiCo-S electrodes were used as anodes in atmospheric pressure AEM-WE single cells fed with 1 M KOH and operating at 60 °C in a dry cathode configuration. Pt/C electrodes (using Aemion+ AF3-HWK9-75-X as ionomer) served as benchmark cathodes. Cells were named after their respective anodes. Both cells underwent the same electrochemical routine reported for the AEL configuration. It is important to point out that, in the case of AEM-WEs, the activation procedure (Figure S3 and related discussion) is fundamental to properly hydrate and condition the AEM itself and is indeed generally recommended by the producers. Figure 5a shows the initial polarization curves (and HFR measured at the end of each current step) for NiCo-D (blue) and NiCo-S (red). The traces obtained from the two cells are almost juxtaposed, with a divergence only at current densities higher than 1.5 A cm^–2^, over which NiCo-D performs slightly better than its sprayed counterpart. Interestingly, the HFR of the cell implementing NiCo-S (i.e., the electrode in which the ionomer is present in the catalyst layer) is higher than that recorded for NiCo-D. These results suggest that the presence of the ionomer in the anodic catalyst layer might not be fundamental for the good functioning of an AEM-WE operated with 1 M KOH anolyte. 24 h long CP at 1 A cm^–2^, collected on both cells (Figures S4a and S5a) return consistent results, while post-CP polarization curves (Figures S4b and S5b) confirm the stability of both systems. The electrochemical traces obtained from the AST conducted on both cells are reported in Figure 5b,c. In accordance with fixed current stability tests (Figures S4 and S5), AST demonstrates the robustness of both systems and their similar voltage performance. Similarly, the lower HFR of the deposited electrode is confirmed.

**Figure 5:**
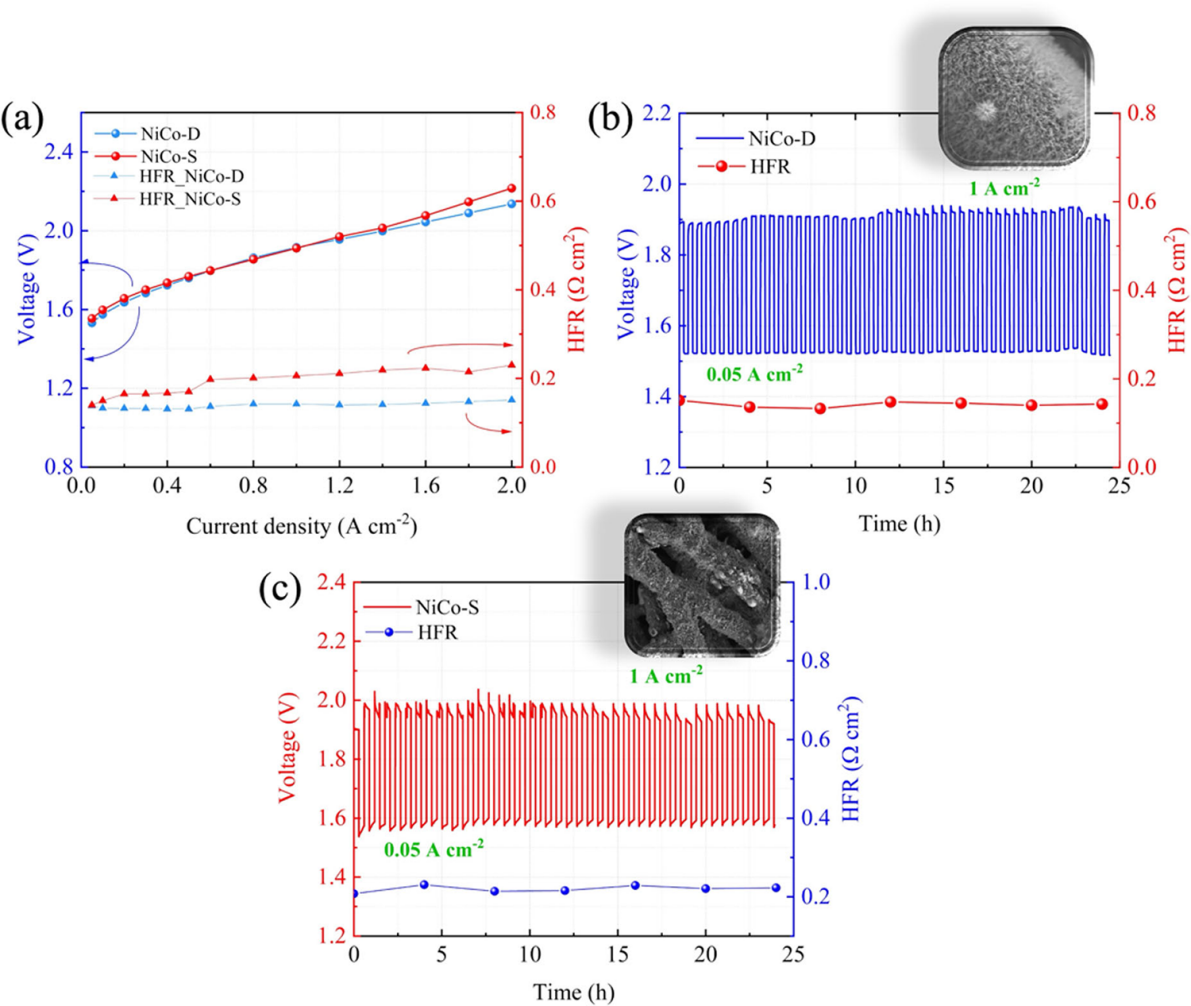
AEM-WE characterization. (a) Polarization curves and HFR collected on NiCo-D and NiCo-S before AST. Electrochemical traces obtained from AST on (b) NiCo-D and (c) NiCo-S.

Post-AST polarization curves (Figure 6a) are consistent with the initial curves, further corroborating the stable performance of the overall electrochemical system. Focusing on anode stability, Figure 6b reports the before and after AST SEM images of both NiCo-D and NiCo-S. For NiCo-D, most nanorods remained intact on the Ni-felt substrate, with some detachment observed (Figure S6, Supporting Information), likely occurring during disassembly, as parts adhered to the membrane. NiCo-S displayed a similar scenario, indicating that the direct growth method produces results comparable to those achieved with the use of binders regarding catalyst adhesion to the electrode surface. The homogeneity of the catalyst layer is preserved on both electrodes, despite a slight roughening of the surface may be qualitatively observed.

**Figure 6:**
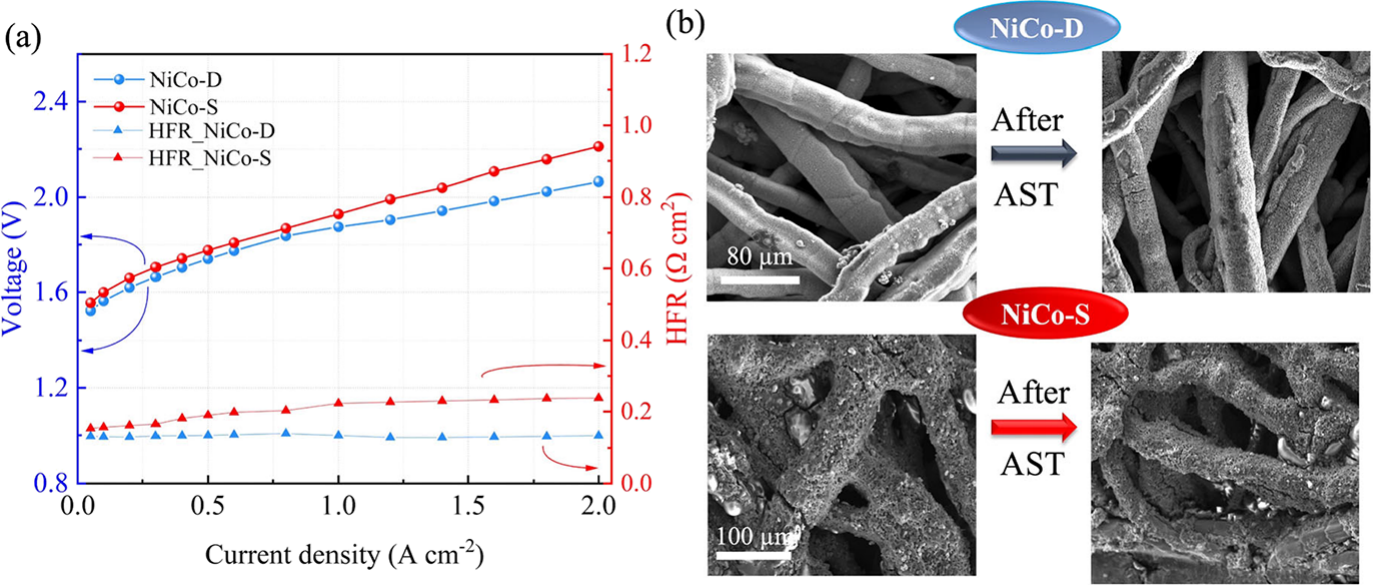
AEM-WE characterization after the AST: (a) polarization curve and HFR of NiCo-D and NiCo-S. (b) SEM images comparing NiCo-D and NiCo-S before and after operation.

When it comes to similar electrodes in this content, the results of both NiCo-D and NiCo-S with 5 mg·cm^–2^ of loading seem reliable for AEM-WE. For instance, Ahmed et al. used the NiCo_2_O_4_ deposited on the nickel foam (25 mg·cm^–2^ of loading) as anode, 1 mg·cm^–2^ of Pt loading on carbon paper in combination with the dioxide membrane Sustainion X-37-50 (grade T with a thickness of 50 μm), which achieved approximately 2.1 V at 1 A·cm^–2^ at 55 °C with 1 M KOH.^54^ Moreover, Cossar et al. which focused on the role of ionomer in the performance of electrode and AEM-WE revealed that the Ni_90_Fe_10_ catalyst with 5 mg·cm^–2^ of loading on gold (Au)-coated titanium (Ti) felt (2GDL20-1,0, 1 mm thick, Bekaert Corporation, Marietta, GA) in combination with 1 mg·cm^–2^ of Pt/C loading on Teflon-treated carbon paper with Aemion AF1-HNN8-50-X membrane achieved 1.941 V at 0.4 A·cm^–2^.^37^

## 4 Conclusions

In this study, we successfully tested a NiCo_2_O_4_ catalyst, modified in morphology using the sono-hydrothermal method, for applications in both AELs and AEM-WEs. Physicochemical analyses, including SEM and TEM, confirmed the nanorod-like structure of the catalyst, which enhances electrochemical activity and improves conductivity. These findings were further supported by three-electrode cell measurements.

Using innovative fabrication methods, we demonstrated the effectiveness of direct-grown NiCo_2_O_4_ electrodes as binder-free solutions for AELs, achieving an outstanding performance (cell voltage of 1.78 V at 1 A cm^–2^). Additionally, we compared the binder-free NiCo_2_O_4_ electrodes with their spray-coated counterparts incorporating ionomers in AEM-WE systems. Both electrode types exhibited promising performances (1.915 V vs 1.911 V at 1 A cm^–2^ after preconditioning) and durability, as validated by 24-h fixed current stability tests followed by 24-h accelerated stress tests.

The binder-free approach, which yielded excellent results in AELs, is therefore also a promising methodology for AEM-WEs, especially considering the safety and environmental benefits of avoiding nanometric powder spraying during electrode fabrication. These results also highlight the versatility, scalability, and sustainability of NiCo_2_O_4_-based electrodes for advanced electrolyzer technologies. Although optimizing ionomer and catalyst loading on the electrode surface by spraying may provide further performance improvements, paving the way for more efficient large-scale electrolyzer applications compared to current designs of AEMWEs and AELs, this preliminary study indicates that the use of directly grown (i.e., binder-free) anodes may represent an efficient platform under both AEL and AEM-WE configurations.

## Supplementary Material



## Notes

Figure S1: voltage and HFR during AEL activation; Figure S2: AEL fixed load stability test; Figure S3: AEM-WE activation; Figure S4: NiCo-D AEM-WE electrochemical stability and performance; Figure S5: NiCo-S AEM-WE electrochemical stability and performance; Figure S6: SEM analysis of NiCo-D single fiber after test in AEM-WE; and Figure S7: SEM image of the bottom surface of the Ni felt after NiCo-D deposition (PDF)

## References

[1] Campo
Schneider L. P., Dhrioua M., Ullmer D., Egert F., Wiggenhauser H. J., Ghotia K., Kawerau N., Grilli D., Razmjooei F., Ansar S. A. (2024). Advancements in Hydrogen Production
Using Alkaline Electrolysis Systems: A Short Review on Experimental
and Simulation Studies. Curr. Opin Electrochem.

[2] Afanasev P., Askarova A., Alekhina T., Popov E., Markovic S., Mukhametdinova A., Cheremisin A., Mukhina E. (2024). An Overview of Hydrogen
Production Methods: Focus on Hydrocarbon Feedstock. Int. J. Hydrogen Energy.

[3] Hassan Q., Algburi S., Sameen A. Z., Salman H. M., Jaszczur M. (2024). Green Hydrogen:
A Pathway to a Sustainable Energy Future. Int.
J. Hydrogen Energy.

[4] Khataee A., Shirole A., Jannasch P., Krüger A., Cornell A. (2022). Anion Exchange Membrane Water Electrolysis
Using Aemion^TM^ Membranes and Nickel Electrodes. J.
Mater. Chem. A Mater..

[5] Klingenhof M., Selve S., Günther C. M., Schmidt J., Razmjooei F., Strasser P., Ansar S.-A. (2024). All Platinum-Group-Metal-Free
Alkaline
Exchange Membrane Water Electrolyzers Using Direct Hydrothermal Catalyst
Deposition on Raney Ni Substrate. ACS Appl.
Energy Mater..

[6] Lira
Garcia Barros R., Kelleners M. H. G., van Bemmel L., van der Leegte T. V. N., van der Schaaf J., de Groot M. T. (2024). Elucidating the
Increased Ohmic Resistances in Zero-Gap Alkaline Water Electrolysis. Electrochim. Acta.

[7] Kim Y., Jung S.-M., Kim K.-S., Kim H.-Y., Kwon J., Lee J., Cho H.-S., Kim Y.-T. (2022). Cathodic Protection System against
a Reverse-Current after Shut-Down in Zero-Gap Alkaline Water Electrolysis. JACS Au.

[8] Jung S., Kim Y., Lee B., Jung H., Kwon J., Lee J., Kim K., Kim Y., Kim K., Cho H., Park J. H., Han J. W., Kim Y. (2024). Reverse-Current Tolerance for Hydrogen
Evolution Reaction Activity of Lead-Decorated Nickel Catalysts in
Zero-Gap Alkaline Water Electrolysis Systems. Adv. Funct. Mater..

[9] Amireh S. F., Heineman N. N., Vermeulen P., Barros R. L. G., Yang D., van der Schaaf J., de Groot M. T. (2023). Impact of Power Supply Fluctuation
and Part Load Operation on the Efficiency of Alkaline Water Electrolysis. J. Power Sources.

[10] Kang S., Kim Y., Wilke V., Bae S., Chmielarz J. J., Sanchez D. G., Ham K., Gago A. S., Friedrich K. A., Lee J. (2024). Stabilizing Pure Water-Fed Anion
Exchange Membrane Water Electrolyzers
through Membrane–Electrode Interface Engineering. ACS Appl. Mater. Interfaces.

[11] de
Groot M. T., Vreman A. W. (2021). Ohmic Resistance in Zero Gap Alkaline
Electrolysis with a Zirfon Diaphragm. Electrochim.
Acta.

[12] Zhu J., Chen W., Poli S., Jiang T., Gerlach D., Junqueira J. R. C., Stuart M. C. A., Kyriakou V., Costa Figueiredo M., Rudolf P., Miola M., Morales D. M., Pescarmona P. P. (2024). Nanostructured
Fe-Doped Ni _3_ S _2_ Electrocatalyst for the Oxygen
Evolution Reaction with High Stability at an Industrially-Relevant
Current Density. ACS Appl. Mater. Interfaces.

[13] Chen B., Mardle P., Holdcroft S. (2022). Probing the Effect of Ionomer Swelling
on the Stability of Anion Exchange Membrane Water Electrolyzers. J. Power Sources.

[14] Moradizadeh L., Madhavan P. V., Chellehbari Y. M., Gupta A., Li X., Shahgaldi S. (2024). Porous Transport
Layers with Low Pt Loading Having
Nb–Ta Alloy as Interlayer for Proton Exchange Membrane Water
Electrolyzers. Int. J. Hydrogen Energy.

[15] Kang Z., Chen Y., Wang H., Alia S. M., Pivovar B. S., Bender G. (2022). Discovering and Demonstrating
a Novel High-Performing
2D-Patterned Electrode for Proton-Exchange Membrane Water Electrolysis
Devices. ACS Appl. Mater. Interfaces.

[16] Falqueto J. B., Hales N., Schimidt T. J., Fabbri E. (2024). Recent Advances in
Nickel-Based Perovskite Oxides for the Electrocatalytic Oxygen Evolution
Reaction in Alkaline Electrolytes. ACS Mater.
Lett..

[17] Galkina I., Faid A. Y., Jiang W., Scheepers F., Borowski P., Sunde S., Shviro M., Lehnert W., Mechler A. K. (2024). Stability of Ni–Fe-Layered Double Hydroxide
Under Long-Term Operation in AEM Water Electrolysis. Small.

[18] Klingenhof M., Trzesniowski H., Koch S., Zhu J., Zeng Z., Metzler L., Klinger A., Elshamy M., Lehmann F., Buchheister P. W., Weisser A., Schmid G., Vierrath S., Dionigi F., Strasser P. (2024). High-Performance Anion-Exchange Membrane
Water Electrolysers Using NiX (X = Fe,Co,Mn) Catalyst-Coated Membranes
with Redox-Active Ni–O Ligands. Nat.
Catal.

[19] Mayerhöfer B., McLaughlin D., Böhm T., Hegelheimer M., Seeberger D., Thiele S. (2020). Bipolar Membrane Electrode
Assemblies
for Water Electrolysis. ACS Appl. Energy Mater..

[20] Hyun J., Hwan Yang S., Wook Lee D., Oh E., Bae H., Suc Cha M., Doo G., Yong Lee J., Kim H.-T. (2023). Impact
of the Binding Ability of Anion Exchange Ionomer on the Initial Performance
Degradation of Anion Exchange Membrane Water Electrolyzers. Chemical Engineering Journal.

[21] Chand K., Paladino O. (2023). Recent Developments
of Membranes and Electrocatalysts
for the Hydrogen Production by Anion Exchange Membrane Water Electrolysers:
A Review. Arabian Journal of Chemistry.

[22] Mardle P., Chen B., Holdcroft S. (2023). Opportunities of Ionomer Development
for Anion-Exchange Membrane Water Electrolysis. ACS Energy Lett..

[23] Omidi-Dargahi A., Dehghani H., Ehsani A. (2024). Electrochemical
Performance of NiCo2O4/Functionalized
Graphene Oxide with Phenylalanine and Tryptophane as Efficient Electrodes
to Enhance Capacitance Properties in Supercapacitors. J. Energy Storage.

[24] Kannangara Y. Y., Karunarathne S., Wijesinghe W. P. S. L., Sandaruwan C., Ratwani C. R., Kamali A. R., Abdelkader A. M. (2024). The Electrochemical
Performance of Various NiCo2O4 Nanostructures in Hybrid Supercapacitors:
Investigating the Impact of Crystalline Defects. J. Energy Storage.

[25] Perroni P. B., Ferraz T. V. B., Rousseau J., Canaff C., Varela H., Napporn T. W. (2023). Stainless Steel Supported NiCo2O4
Active Layer for
Oxygen Evolution Reaction. Electrochim. Acta.

[26] Shi H., Zhao G. (2014). Water Oxidation on
Spinel NiCo _2_ O _4_ Nanoneedles
Anode: Microstructures, Specific Surface Character, and the Enhanced
Electrocatalytic Performance. J. Phys. Chem.
C.

[27] Paladino O., Niyati A., Moranda A., Beigzadeh Arough P., Marcenaro B. (2025). Engineering Potential Electrocatalysts for Both AEM
Electrolyzers and Redox Flow Batteries: Design of Experiments at the
Different Scales. Appl. Therm Eng..

[28] Niyati A., Moranda A., Beigzadeh
Arough P., Navarra F. M., Paladino O. (2024). Electrochemical
Performance of a Hybrid NiCo2O4@NiFelt Electrode at Different Operating
Temperatures and Electrolyte PH. Energies (Basel).

[29] Agudosi E. S., Abdullah E. C., Numan A., Mubarak N. M., Aid S. R., Benages-Vilau R., Gómez-Romero P., Khalid M., Omar N. (2020). Fabrication
of 3D Binder-Free Graphene NiO Electrode for Highly Stable Supercapattery. Sci. Rep.

[30] Wang L., Weissbach T., Reissner R., Ansar A., Gago A. S., Holdcroft S., Friedrich K. A. (2019). High Performance
Anion Exchange Membrane
Electrolysis Using Plasma-Sprayed, Non-Precious-Metal Electrodes. ACS Appl. Energy Mater..

[31] Koch S., Heizmann P. A., Kilian S. K., Britton B., Holdcroft S., Breitwieser M., Vierrath S. (2021). The Effect of Ionomer Content in
Catalyst Layers in Anion-Exchange Membrane Water Electrolyzers Prepared
with Reinforced Membranes (Aemion+^TM^). J. Mater. Chem. A Mater..

[32] Plevová M., Hnát J., Žitka J., Pavlovec L., Otmar M., Bouzek K. (2022). Optimization
of the Membrane Electrode Assembly for
an Alkaline Water Electrolyser Based on the Catalyst-Coated Membrane. J. Power Sources.

[33] Fortin P., Khoza T., Cao X., Martinsen S. Y., Oyarce Barnett A., Holdcroft S. (2020). High-Performance
Alkaline Water Electrolysis
Using Aemion^TM^ Anion Exchange Membranes. J. Power Sources.

[34] Niyati A., Moranda A., Basbus J. F., Paladino O. (2024). Unlocking the Potential
of NiCo _2_ O _4_ Nanocomposites: Morphology Modification
Based on Urea Concentration and Hydrothermal and Calcination Temperature. New J. Chem..

[35] Qiu C., Xu Z., Chen F.-Y., Wang H. (2024). Anode Engineering for Proton Exchange
Membrane Water Electrolyzers. ACS Catal..

[36] Park J. E., Bae H. E., Karuppannan M., Oh K. M., Kwon O. J., Cho Y.-H., Sung Y.-E. (2022). Effect of Catalyst Layer Designs
for High-Performance and Durable Anion-Exchange Membrane Water Electrolysis. Journal of Industrial and Engineering Chemistry.

[37] Cossar E., Murphy F., Walia J., Weck A., Baranova E. A. (2022). Role of
Ionomers in Anion Exchange Membrane Water Electrolysis: Is Aemion
the Answer for Nickel-Based Anodes?. ACS Appl.
Energy Mater..

[38] Volk E. K., Kreider M. E., Kwon S., Alia S. M. (2024). Recent Progress
in Understanding the Catalyst Layer in Anion Exchange Membrane Electrolyzers–Durability,
Utilization, and Integration. EES Catalysis.

[39] Faid A. Y., Xie L., Barnett A. O., Seland F., Kirk D., Sunde S. (2020). Effect of
Anion Exchange Ionomer Content on Electrode Performance in AEM Water
Electrolysis. Int. J. Hydrogen Energy.

[40] Vincent I., Lee E.-C., Kim H.-M. (2020). Highly Cost-Effective Platinum-Free
Anion Exchange Membrane Electrolysis for Large Scale Energy Storage
and Hydrogen Production. RSC Adv..

[41] Riemer M., Duval-Dachary S., Bachmann T. M. (2023). Environmental Implications of Reducing
the Platinum Group Metal Loading in Fuel Cells and Electrolysers:
Anion Exchange Membrane versus Proton Exchange Membrane Cells. Sustainable Energy Technologies and Assessments.

[42] Chen N., Paek S. Y., Lee J. Y., Park J. H., Lee S. Y., Lee Y. M. (2021). High-Performance
Anion Exchange Membrane Water Electrolyzers
with a Current Density of 7.68 A Cm ^–2^ and a Durability
of 1000 h. *Energy*. Environ.
Sci..

[43] Kawashima K., Márquez R. A., Son Y. J., Guo C., Vaidyula R. R., Smith L. A., Chukwuneke C. E., Mullins C. B. (2023). Accurate Potentials
of Hg/HgO Electrodes: Practical Parameters for Reporting Alkaline
Water Electrolysis Overpotentials. ACS Catal..

[44] Kou T., Wang S., Li Y. (2021). Perspective on High-Rate Alkaline
Water Splitting. ACS Mater. Lett..

[45] Xu D., Stevens M. B., Cosby M. R., Oener S. Z., Smith A. M., Enman L. J., Ayers K. E., Capuano C. B., Renner J. N., Danilovic N., Li Y., Wang H., Zhang Q., Boettcher S. W. (2019). Earth-Abundant
Oxygen Electrocatalysts for Alkaline
Anion-Exchange-Membrane Water Electrolysis: Effects of Catalyst Conductivity
and Comparison with Performance in Three-Electrode Cells. ACS Catal..

[46] Yang G., Park S.-J. (2018). Facile Hydrothermal Synthesis of
NiCo2O4- Decorated
Filter Carbon as Electrodes for High Performance Asymmetric Supercapacitors. Electrochim. Acta.

[47] Senthil R. A., Jung S., Min A., Kumar A., Moon C. J., Singh M., Choi M. Y. (2024). Revealing
the Impact of Pulsed Laser-Produced
Single-Pd Nanoparticles on a Bimetallic NiCo _2_ O _4_ Electrocatalyst for Energy-Saving Hydrogen Production via Hybrid
Water Electrolysis. ACS Catal..

[48] Waghmode R. B., Maile N. C., Lee D. S., Torane A. P. (2020). Chemical Bath Synthesis
of NiCo2O4 Nanoflowers with Nanorods like Thin Film for Flexible Supercapacitor
Application-Effect of Urea Concentration on Structural Conversion. Electrochim. Acta.

[49] Liu Z.-Q., Xu Q.-Z., Wang J.-Y., Li N., Guo S.-H., Su Y.-Z., Wang H.-J., Zhang J.-H., Chen S. (2013). Facile Hydrothermal
Synthesis of Urchin-like NiCo2O4 Spheres as Efficient Electrocatalysts
for Oxygen Reduction Reaction. Int. J. Hydrogen
Energy.

[50] Chang C., Zhang L., Hsu C.-W., Chuah X.-F., Lu S.-Y. (2018). Mixed NiO/NiCo _2_ O _4_ Nanocrystals Grown from the Skeleton of a
3D Porous Nickel Network as Efficient Electrocatalysts for Oxygen
Evolution Reactions. ACS Appl. Mater. Interfaces.

[51] Sha L., Ye K., Wang G., Shao J., Zhu K., Cheng K., Yan J., Wang G., Cao D. (2019). Hierarchical NiCo2O4 Nanowire Array
Supported on Ni Foam for Efficient Urea Electrooxidation in Alkaline
Medium. J. Power Sources.

[52] Zuo Y., Bellani S., Saleh G., Ferri M., Shinde D. V., Zappia M. I., Buha J., Brescia R., Prato M., Pascazio R., Annamalai A., de Souza D. O., De Trizio L., Infante I., Bonaccorso F., Manna L. (2023). Ru–Cu Nanoheterostructures
for Efficient Hydrogen Evolution Reaction in Alkaline Water Electrolyzers. J. Am. Chem. Soc..

[53] Mardle P., Chen B., Liu H., Xie Z., Qu W., Holdcroft S. (2025). Effects of Aemion and Aemion+ Binders
in Oxygen Evolution
Reaction Catalyst Layers. Electrochim. Acta.

[54] Ahmed K. W., Habibpour S., Chen Z., Fowler M. (2024). Investigation of NiCoOx
Catalysts for Anion Exchange Membrane Water Electrolysis: Performance,
Durability, and Efficiency Analysis. J. Energy
Storage.

