# Supplementary material for: Electrochemical Performance of Engineered NiCo2O4 in AEM Water Electrolyzers: Direct-Growth vs Spray-Coated Anode
Source: ACS Appl Energy Mater. 2025 Aug 5;8(16):12039–48. doi: 10.1021/acsaem.5c01487 (PMC12381872; doi:10.1021/acsaem.5c01487)
Supplement: Supplementary file 1 [file ae5c01487_si_001.pdf]

Supporting Information

Electrochemical Performance of Engineered $NiCo_2O_4$ in AEM Water Electrolysers: Direct-Growth vs. Spray-Coated Anode

Ataollah Niyati[1], Arianna Moranda[1], Sebastiano Bellani[2], Thi Hong Hanh Le[3], Michele Ferri[2,3], Ombretta Paladino[1]*

1. Department of Civil, Chemical and Environmental Engineering (DICCA), Università di Genova, Via Opera Pia 15, 16145 Genoa, Italy

2. Antares Electrolysis S.r.l., Piazza della Vittoria, 14/19, 16121 Genova, Italy

3. Nanochemistry Department, Istituto Italiano di Tecnologia (IIT) , Via Morego 30, 16163, Genova, Italy

*Corresponding Authors:
Ombretta Paladino: Paladino@unige.it

1. Pseudo-Tafel slope vs. Tafel slope

The Tafel slopes presented in this work were extracted from linear sweep voltammetry (LSV) curves recorded at a scan rate of 5 mV/s. Since the potential was continuously varied during the measurements, the electrodes were not allowed sufficient time to reach full steady-state conditions at each potential step although this is the main method used in majority of studies. According to Fletcher et al. [1], a true Tafel slope should be obtained under strict steady-state conditions to eliminate contributions from capacitive currents, mass transport limitations, and transient phenomena, while this is hard for a non-ideal sample like NiCo-S and NiCo-D. As the LSV method inherently involves dynamic conditions, the extracted slopes are considered to be pseudo-Tafel slopes. To be more precise, although the values provide useful comparative information on the electrocatalytic behavior of direct-grown and sprayed electrodes, they do not fully represent steady-state kinetic parameters while allowing for a qualitative comparison of the reaction kinetics between the two types of electrode fabrication methods.

2. AEL Activation Process

A combination of activation processes is conducted before testing the AEL. The electrolyte was pumped to the AEL at a flow rate of 5 mL $min^{-1}$and the cell temperature was adjusted to 80°C (monitored through a K-type thermocouple) through the combination of electrolyte heating (reservoirs placed in oil baths) and direct cell heating. Fig S1 shows the activation process for

NiCo-D in AEL configuration, starting with a 40 minutes-long galvanostatic step at 200 mA cm^{-2} followed by a 2-hour-long step at 1 A cm^{-2}, while continuously measuring the HFR.

The I-V curve of the cell stabilizes in the last 30-40 minutes of the activation process. Accordingly, the HFR of the system reaches a stable minimum value at *ca.* 0.1 Ω cm^2, also confirming the stabilization (and thermalization) of the cell.

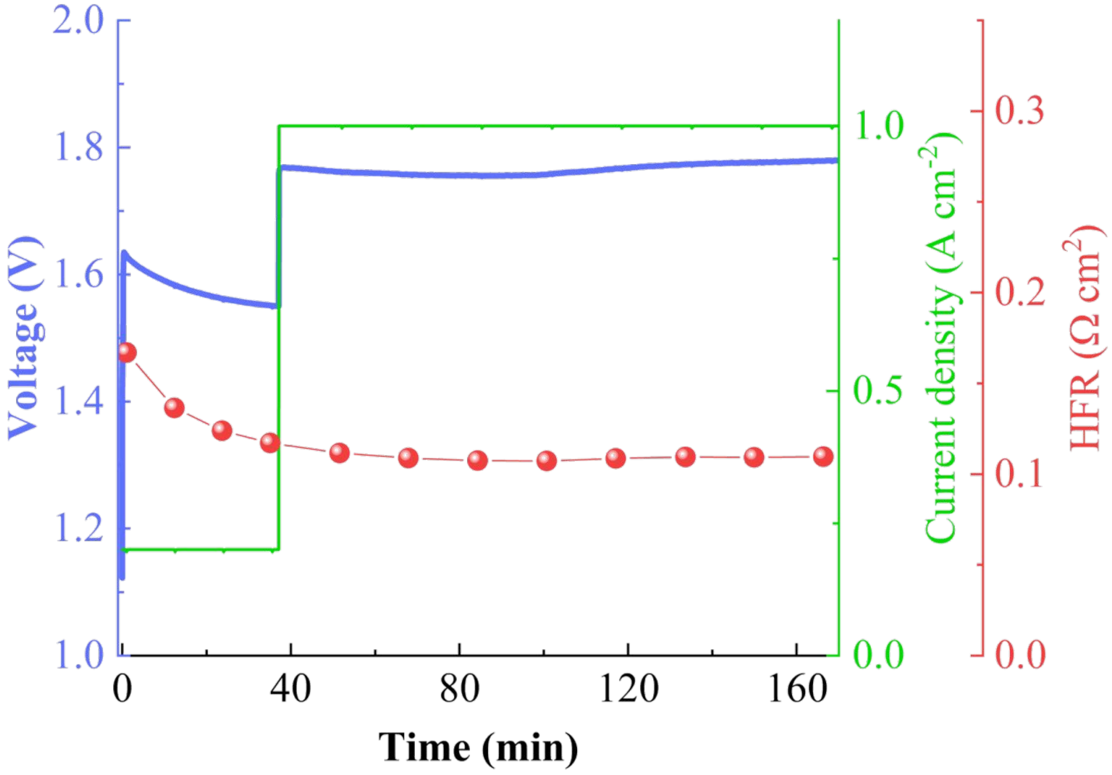

Figure S 1. Voltage and HFR recorded during the activation procedure of the AEL cell. The procedure consisted in two steps: a 40 minutes long CP @ 200 mA cm^{-2} followed by a *ca.* 150 minutes long CP @ 1 A cm^{-2}. HFR is measured by EIS, carried out each 10 minutes during both CPs.

3. AEL 24 hr-DC Stability test

After the activation process, a polarization curve was measured, and a 24-h DC test as a durability test was performed at 1 A cm^{-2} for NiCo-D, which results are depicted in Figure S2.

The cell voltage (registered at 1 A cm^{-2}) for the AEL cell implementing the NiCo-D electrode as anode was 1.747 V (HFR 0.102 Ω cm^2) aat the beginning of the scan and 1.780 V (HFR 0.101 Ω cm^2) at the end, revealing a robust and stable performance of the system.

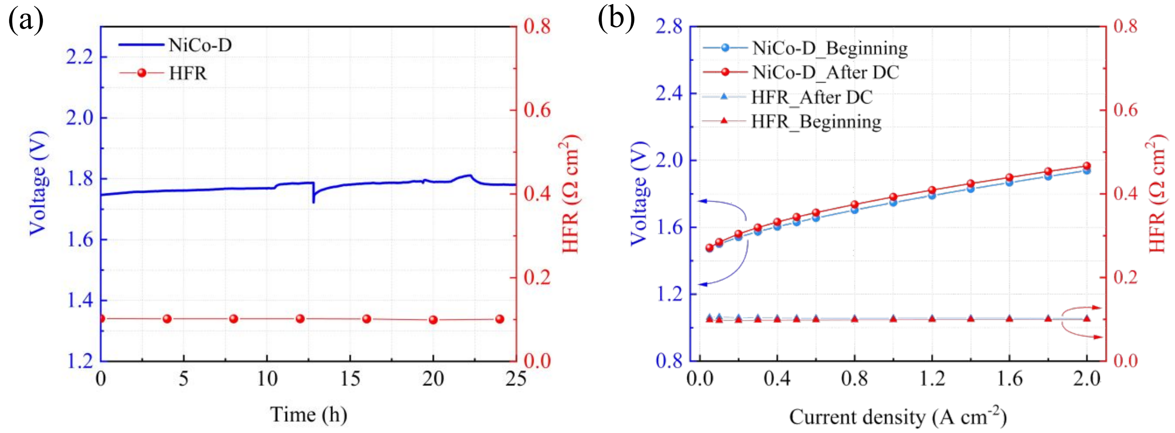

Figure S 2. AEL fixed load stability test: (a) a 24-hour-long CP @ 1 A cm^{-2} by measuring HFR every 4 hours by EIS. (b) Polarization curve comparison before and after a 24-hour-long CP test by measuring HFR every 4 hours by EIS.

4. AEM-WE Activation Process

A sample activation of the AEM-WE (the one implementing NiCo-D as anode) is reported in Figure S3. Comparing the outcomes of this activation procedure with those obtained for the same electrodes but under AEL configuration (Figure S1), a few differences can be noted. First, the cell voltage (at 1 A cm^{-2}) of the AEM-WE (1.91 V) is higher than that recorder under AEL

configuration (1.743 V). Consistently, a higher HFR is measured in the former (0.159 Ω cm^2 in AEM-WEs, 0.100 Ω cm^2 in AEL). On the other hand, the I-V curves and HFR trend plateaus faster under AEM-WEs configuration, indicating a quicker conditioning and, allegedly, a quicker cold start-up of this cell in comparison with the typical AEL configuration.

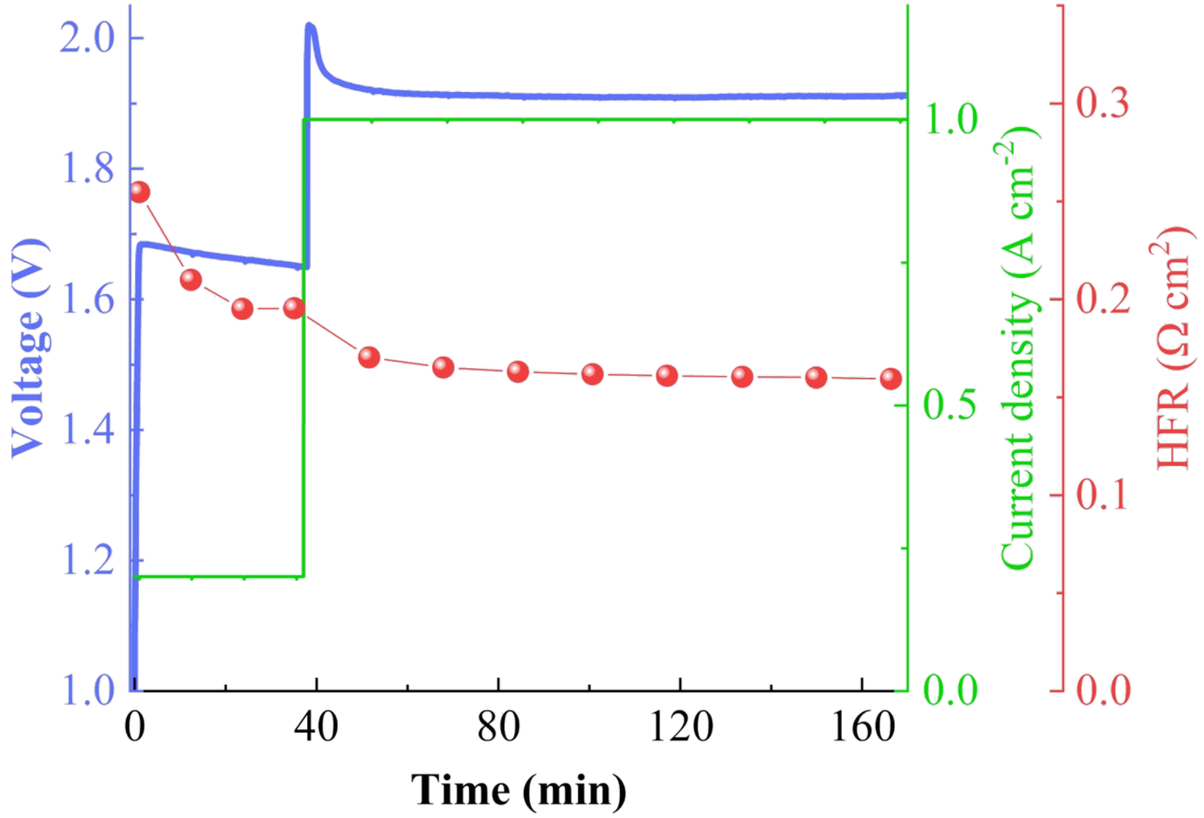

Figure S 3. Sample AEM-WE activation (cell implementing NiCo-D as anode). The same conditions described in Figure S1 hold for this test.

5. AEM-WE 24 hr-DC Stability test

Figure S4 and Figure S5 report the 24 hours long CP (at 1 A cm^{-2}) and post-CP polarization curves collected on both AEM-WEs cells. For NiCo-D, the cell voltage stabilized around 1.93 V at the end of the test, with a constant HFR of *ca.* 0.154 Ω cm^2 (Figure S4a). Variability of both voltage and HFR throughout the test was minimal, and complete retention of performance was confirmed by the perfect superimposition of pre- and post-CP polarization curves (Figure S4b). NiCo-S returned a mostly constant cell voltage of *ca.* 1.94 V with a stable HFR of 0.207 Ω cm^2.

In this case, a slight (overall negligible) decrease in the performance of the cell was assessed by comparing pre-and post-polarization curves (Figure S5b). Overall, NiCo-D and NiCo-S perform similarly in terms of cell voltage at the same delivered current. However, in agreement with the results reported in Figure 5a, a lower HFR is obtained for NiCo-D.

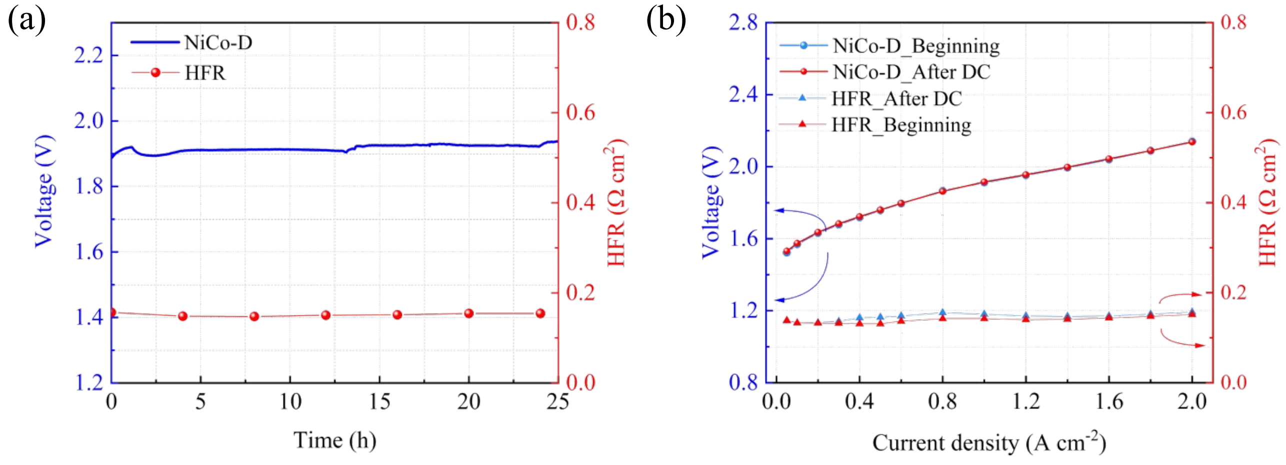

Figure S 4. NiCo-D AEM-WE electrochemical stability and performance. (a) Fixed load stability test, carried out by means of a 24-hours long CP @ 1 A cm^{-2}. HFR is measured every 4 hours by EIS. (b) Pre- and post-CP polarization curves (with HFR measured at the end of each galvanostatic.

step).

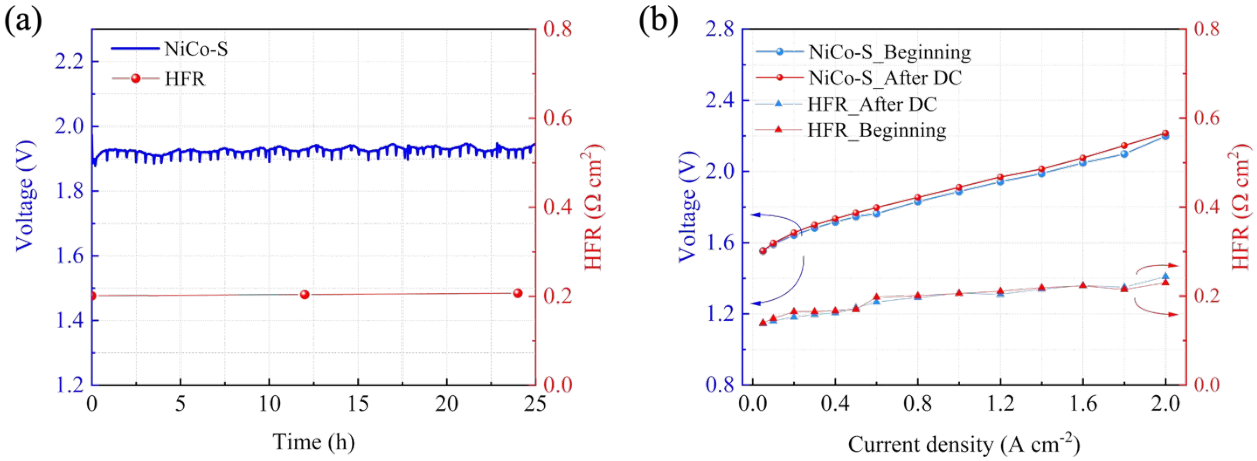

Figure S 5. NiCo-S AEM-WE electrochemical stability and performance. (a) Fixed load stability test, carried out by means of a 24-hour-long CP @ 1 A cm^{-2}. HFR is measured every 4 hours by EIS. (b) Pre- and post-CP polarization curves (with HFR measured at the end of each galvanostatic step).

6. Additional SEM analysis of NiCo-D

7.1 NiCo-D after AEM-WE test

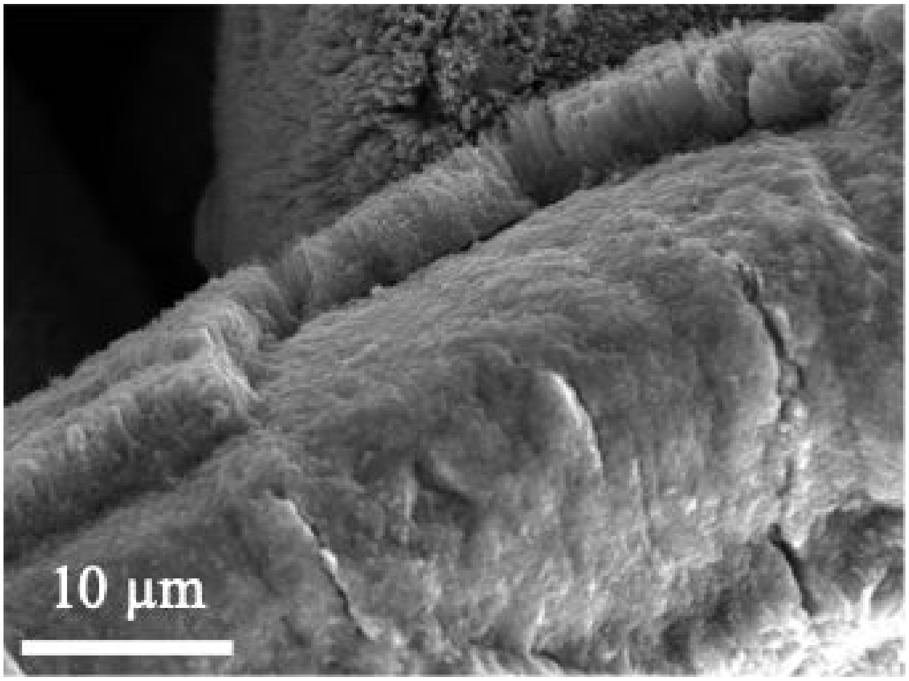

Figure S 6. SEM analysis of NiCo-D single Fiber showing a detachment of nanorods from the surface, after test in AEM-WE.

7.1 NiCo-D coating on the bottom surface of Nifelt

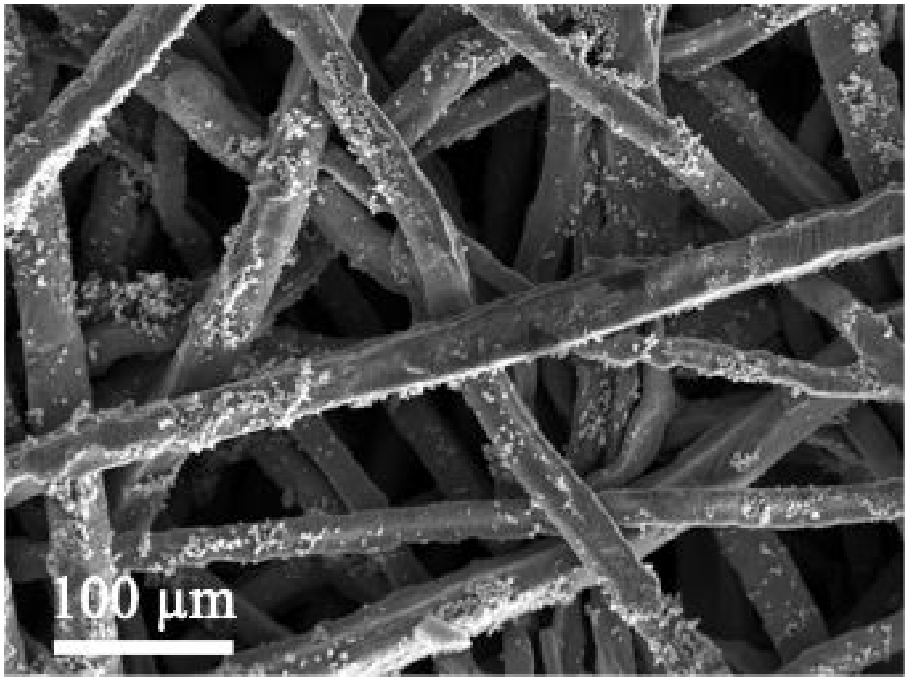

Figure S 7. SEM image of the bottom surface of the Ni felt substrate after deposition of NiCo-D.

References

(1) Fletcher, S. Tafel Slopes from First Principles. *Journal of Solid State Electrochemistry* 2009, *13* (4), 537–549. https://doi.org/10.1007/s10008-008-0670-8.
